# Molecular detection of *Leishmania infantum* DNA and host blood meal identification in *Phlebotomus* in a hypoendemic focus of human leishmaniasis in northern Algeria

**DOI:** 10.1371/journal.pntd.0006513

**Published:** 2018-06-29

**Authors:** Kahina Bennai, Djamel Tahir, Ismail Lafri, Amina Bendjaballah-Laliam, Idir Bitam, Philippe Parola

**Affiliations:** 1 Aix Marseille Univ, IRD, AP-HM, SSA, VITROME, IHU Méditerranée Infection, Marseille, France; 2 Laboratoire de Valorisation et Conservation des Ressources Biologiques (VALCOR), Faculté des Sciences, Université M’Hamed Bougara, Boumerdes, Algérie; 3 Institut des Sciences Vétérinaires, Université Blida 1, Blida, Algérie; 4 Etablissement Public Hospitalier de Hadjout, Tipaza, Algérie; 5 Laboratoire Biodiversité et Environnement, Université des Sciences et Technologies Houari Boumediene, Alger, Algérie; 6 Ecole Supérieure des Sciences de l'Aliment et des Industries Agro-Alimentaires, Alger, Algérie; Charité University Medicine Berlin, GERMANY

## Abstract

**Background:**

*Leishmania* parasites are transmitted by female phlebotomine sand flies that maintain the enzootic cycle by circulating between sylvatic and domestic mammals. Humans are part of this cycle as accidental hosts due to the vector’s search for a source of blood. In Algeria, Human Leishmaniases (HL) are endemic and represent a serious public health problem because of their high annual incidence and their spread across the country. The aim of this study is to identify sand fly species fauna (vectors of *Leishmania*), determine their infection rate and identify their feeding preferences using molecular tools in a hypoendemic focus of HL located in the province of Tipaza, northern Algeria.

**Methodology/Principal findings:**

An entomological survey using CDC light traps was conducted between July and October of 2015 in four HL affected peri-urban locations in the province of Tipaza, northern Algeria. Sand flies were identified using the morphological criteria of the genitalia for the males and spermathecae for the females. *Leishmania* DNA was detected in pooled female sand flies (N = 81 pools with 8–10 specimens per pool) using quantitative real-time polymerase chain reaction (qPCR) targeting two different genes: kDNA-PCR and 18S rRNA. To identify their blood meal sources, blood-fed female sand flies were analyzed by PCR-sequencing targeting the vertebrate cytochrome c oxidase I (*COI*) gene. A total of 4,045 sand flies were caught, of which 3,727 specimens were morphologically identified. Seven species were recorded: *P*. *(L*.*) perniciosus* (50.28%), *P*. *(L*.*) perfiliewi* (26.13%), *P*. *(L*.*) longicuspis* (21.92%), *Sergentomyia (S*.*) minuta* (0.85%), *P*. *(P*.*) papatasi* (0.42%), *P*. *(L*.*) langeroni* (0.32%) and *P*. *(L*.*) ariasi* (0.05%). Afterwards, 740 female specimens were randomly selected and divided into 81 pools and were then screened to investigate the presence of *Leishmania* spp. *L*. *infantum* DNA was detected in three pools, corresponding to three sand fly specimens (one each). The infection rate was 0.33% (2/600) for *P*. *(L*.*) perniciosus* and 2.56% (1/39) for *P*. *(L*.*) perfiliewi*. Analysis of the blood feeding sources (N = 88 specimens) revealed that sand flies belonging to *Larroussius* subgenera, mainly (71.5%) feed on small ruminants. Human blood is the second feeding source (17%), eight specimens (9%) were found to feed on equines and no domestic reservoir (dog) blood was found.

**Conclusions/Significance:**

The presence of human leishmaniasis cases, the high abundance of *Phlebotomus (Larroussius)* species which are proven or suspected vectors of *L*. *infantum*, and the detection of *L*. *infantum* DNA from its natural vectors (*P*. *(L*.*) perniciosus*, *P*. *(L*.*) perfiliewi*), in addition to the blood-feeding of positive females for *L*. *infantum* on humans blood, prove that the major elements of the epidemiological transmission cycle of *L*. *infantum* are present and indicate risk factors for an outbreak of the disease in the province of Tipaza.

## Author summary

Leishmaniases are a group of diseases with various clinical manifestations caused by parasites (*Leishmania* spp.). More than 20 *Leishmania* species that affect human and mammals are transmitted by the bite of an infected female phlebotomine sand flies. In Algeria, two main forms of the disease exist: visceral leishmaniasis (VL) and cutaneous leishmaniasis (CL). Although CL is the most common form of the disease, VL is the most serious and can be fatal if untreated. The purpose of the study was to investigate sand fly species fauna, their infection rate and feeding preference determination in a hypoendemic focus of human leishmaniasis located in the province of Tipaza, northern Algeria using molecular techniques. Seven phlebotomine sand flies species were recorded in this study. In addition, natural infection by *L*. *infantum* was confirmed in two sand flies species (*P*. (*L)*. *perniciosus* and *P*. *(L)*. *perfiliewi*). Human blood was the second (17%) feeding source after ruminants (74.11%%).

## Introduction

Leishmaniases are neglected vector-born tropical diseases caused by more than twenty parasite species belonging to the *Leishmania* genus (Kinetoplastida: Trypanosomatidae) and transmitted to humans by the bite of Phlebotominae (Diptera: Psychodidae) sand flies [1]. *Leishmania* parasites have a digenetic life cycle, alternating between mammalian hosts, including humans, and female sand fly vectors belonging to the *Phlebotomus* genus in the Old World and the *Lutzomyia* genus in the New World [2]. Around 10% of the 800 sand fly species recorded to date are suspected or proven vectors for the transmission of leishmaniases [3]. According to the World Health Organization (WHO), these infections occur around the world, with more than 98 countries being affected including those around the Mediterranean. Around 350 million people are considered at risk, with 1.3 million new cases and around 30,000 deaths recorded annually [4,5].

In Algeria, human and canine leishmaniasis are endemic. Algeria is considered to be one of the ten most affected countries with a higher prevalence of cutaneous (CL) than visceral leishmaniasis (VL) [5]. Thus, HL in this country represents a serious public health problem and more than seven million people are at risk of infection [3,6]. To date, 24 species of sand flies have been diagnosed in Algeria, including two genera and seven subgenera, of which five sand fly species are proven or suspected vectors [7,8]. Three *Leishmania* species are responsible for the disease in the country. The first is *L*. *major*, the agent of zoonotic cutaneous leishmaniasis (ZCL) transmitted by *P*. *(P*.*) papatasi* [7]. The wild rodents, *Psammomys obesus* and *Meriones shawi* are the main reservoir hosts of this species [6]. ZCLs are mostly recorded in the Sahara and the High Plains with an annual number varied between 13,749 in 2003 to 16,585 in 2011, making therefor an average of 14,752 cases per year [9]. The second is *L*. *infantum*, transmitted by *P*. *(L*.*) perniciosus*, *P*. *(L*.*) perfiliewi* and probably by *P*. *(L*.*) longicuspis*. *L*. *infantum* is mostly located in northern parts of the country. It is noteworthy that *Leishmania* species reported in this region are the causes of two forms of leishmaniasis: sporadic cutaneous leishmaniasis, due to *L*. *infantum* MON-24 and visceral leishmaniasis (VL) due to *L*. *infantum* MON-1, with an average incidence of 200 and 150 cases per year, respectively [10–14]. For both parasite zymodemes, canids are considered to be the main reservoir. The third species is *L*. *killicki*, which belongs to the *L*. *tropica* complex that causes CL. Transmitted by *P*. *(P*.*) sergenti*. *L*. *killicki* has recently been reported in several parts of Algeria (Ghardaia, Annaba, Tipaza) and is generally sympatric with *L*. *major* [15–19]. Regarding this species, the suspected reservoir host is *Masouretiera mzabi*, a rodent close to the *Ctenodactylus gundii* that has been found naturally infected with *L*. *killicki* in Tunisia [20]. The annual incidence remains unknown but is estimated to cause less than 100 cases per year [6].

Epidemiological investigations and entomological surveys have always been crucial to better understand endemicity of leishmaniasis foci as well as determining the relationship between the vector species and the reservoirs involved in the wild transmission cycle of *Leishmania* [21,22]. Studying the blood-feeding pattern of phlebotomine sand flies and their infection status in endemic areas is important. Indeed, such studies enable the identification of the potential mammalian reservoirs and vector feeding preferences [22]. In Algeria, epidemiological surveillance of leishmaniasis infections was generally based on serological assays for human and canine leishmaniases, while vector incrimination was carried out by dissecting freshly caught or cryopreserved specimens for isolation and isoenzymatic characterization of *Leishmania* strains present in their midguts [10,11,23,24]. Recently, many PCR-based molecular approaches with a high degree of sensitivity and specificity have proven to be useful in species detection and identification of *Leishmania* parasites in sand fly vectors. Indeed, PCR has been used for the molecular xenomonitoring of leishmaniasis as well as for determining the feeding behaviors of sand flies [2].

In this study, an epidemiological investigation of leishmaniasis was conducted in a new hypoendemic focus of HL, located in the province of Tipaza, northern Algeria. In this area, human cutaneous leishmaniasis cases due to *L*.*killicki* were clinically suspected and interestingly confirmed by molecular tools for the first time in 2013 [16]. An entomological study combined with molecular tools was used to update informations about the sand flies species present in this area. It also enabled us to determine their infection rate with *Leishmania* spp. and identify their blood meal sources. These data gathered are an important element in order to establish a reliable control program against HL in this area.

## Materials and methods

### Ethical considerations

A verbal informed consent was obtained from homeowners (private or residential areas) were sand fly collection was performed.

### Study area

An entomological investigation was conducted in four periurban locations (Bourkika, Bouyerssane, Sidi Boufadhel and Hadjout) in the province of Tipaza located in northern Algeria (36° 52’ 00”N; 6° 54’ 00” E).

The region of Tipaza includes mountains, hills and plains. Its climate is typically Mediterranean with an annual rainfall of 600 mm, while the temperature ranges between 33°C in summer and 5°C in winter. The characteristics of the soil, adequate for traditional cultivation and farming, in addition to socioeconomic living conditions makes the area suitable for the development of phlebotomine sand flies. The presence of peridomestic animals (*i*.*e*., dogs and rodents) which are the main reservoirs of *Leishmania* spp. is also notable.

### Sand fly collection and morphological identification

Phlebotomine sand flies were collected monthly between July and October 2015, their active season. CDC miniature light traps (John W. Hock Co., Gainesville, FL, USA) were placed twice a week on each site, inside and around human habitations and animal shelters. The traps were set at sunset and left in operation from 6pm to 7am. All sand flies that were captured were removed from the traps and placed into a freezer. Finally, males, blood-fed females and unfed females were sorted and transferred to 3 mL vials (with around 30 specimens each), containing 70% ethanol until morphological identification and DNA extraction.

Taxonomical identification of the collected sand flies was carried out on the basis of the morphological criteria described by Abonnenc *et al*. (1972) and Dedet *et al*. (1984) [7,25]. Identification was based according to the morphology of the male genitalia and female spermathecae. Briefly, all specimens were individually transferred to a glass slide and were dissected by removing the posterior third segment of the abdomen using sterile micro-needles. Dissected segments were cleared in Marc-André solution following the protocols previously described [3], and mounted under a cover slip for microscopic identification using a stereomicroscope (Stereo Zoom Microscope Axio Zoom.V16, Germany). The remainder of the body was preserved at -20°C for further molecular analysis.

### DNA extraction

After morphological identification, the remaining parts of each specimen were digested in 180 μl of G2 lysis buffer (Qiagen Hilden, Germany) and 20 μL of proteinase K (20 mg/mL; Qiagen, Hilden, Germany), incubated overnight at 56°C to ensure complete lysis of the tissue and then the whole DNA was extracted from the mixture in 100 μL of Tris EDTA (TE) buffer using the EZ1 DNA Tissue kit (Qiagen, Hilden, Germany) according to the manufacturer's instructions. The DNA was stored at –20°C until molecular analysis.

### Sand flies molecular screening for *Leishmania* infection

For this epidemiological investigation, we used a molecular tool to screen phlebotomine sand flies collected from one specific site in Bourkika, where four cases of HVL have been recorded in 2014. A significant number of specimens from unfed (N = 284) and blood-fed females (N = 456) were randomly selected and subjected to qPCR analysis for the detection of any specimens containing *Leishmania* spp. DNA.

We first screened the DNA of 740 sand flies divided into 81 monospecific pools (around nine sand flies’ DNA per pool), to investigate the presence of *Leishmania* spp. Sand flies’ DNAs were screened by qPCR, using genus-specific primers and probes targeting the18S rRNA *Leishmania* gene (Table 1) [26]. To identify *Leishmania* species from the 18S qPCR-positive pools, a second qPCR using species-specific primers and probes targeting the *L*. *infantum* kinetoplastic gene was performed (Table 1) [27]. Finally, DNAs from sand flies grouped into each positive pool were analyzed individually to determine the number of positive specimens in each pool and in order to confirm the parasite species using the same primers and probes (Table 1).

**Table 1:** Primers and probes used for real time PCR and conventional PCR in this study.

Target genes	Name	Sequences 5’-3’	Annealing temperature (C°)	Reference
*Leishmania* spp. 18S rRNA gene	Leish_F	ACAAGTGCTTTCCCATCG	60	[26]
	Leish_R	CCTAGAGGCCGTGAGTTG		
	Leish_P	FAM- CGGTTCGGTGTGTGGCGCC-TAMRA		
*L*. *infantum* kinetoplast DNAgene	Leishk_F	CTTTTCTGGTCCTCCGGGTAGG	54	[27]
	Leishk_R	CCACCCGGCCCTATTTTACACCAA		
	Leishk_P	FAM-TTTTCGCAGAACGCCCCTACCCGC-TAMRA		
Vertebrate DNA Cytochrome C Oxidase I gene (vCOI)	vCOI_long F	AAGAATCAGAATARGTGTTG	40	[28]
	vCOI_long R	AACCACAAAGACATTGGCAC		
*Phlebotomus* Cytochrome B Cyt b gene	Phleb_CytB-F	GGAGGAGTAATTGCHCTWGTHAT	45	[29]
	Phleb_CytB-R	AGATATTTACCTKCWTCTTTRTGTTT		

qPCR was carried out in 96-well PCR plates and performed on a CFX96 Touch detection system (Bio-Rad, Marnes-la-Coquette, France) using the Takyon Master Mix according to the manufacturer’s instructions. Each amplification run contained positive controls to test the specific conditions of the reagents (DNA extracted from *L*. *infantum* promastigotes) and negative control (DNA-free water) to detect any possible contamination during preparation of the mix. Samples with a cycle threshold level < 35 were considered positive.

### Identification of sand flies’ blood-feed sources

In order to identify the blood meal sources of the phlebotomine sand flies which were collected in Bourkika, the DNA from a total of 88 totally- and partially-engorged female specimens was randomly selected: *P*. *perniciosus* (N = 42), *P*. *longicuspis* (N = 28), *P*. *perfiliewi* (N = 17) and *S*. *minuta* (N = 1). DNA amplification was performed by standard PCR using primers targeting the vertebrate cytochrome c oxidase I gene (*COI*), as previously described (Table 1) [28]. PCR products were then sequenced using the Applied Biosystems 3130xl Genetic Analyzer (Thermo Fisher Scientific, France) using the DNA sequencing BigDye Terminator Kit (Perkin-Elmer), according to the manufacturer’s instructions. Finally, the sequences obtained were assembled using ChromasPro 1.7 software (Technelysium Pty Ltd., Tewantin, Australia) and were then compared to those available in the GenBank database using BLAST software (www.ncbi.nlm.nih.gov/BLAST).

### Molecular identification of sand flies

To confirm species identification of *Leishmania* positive sand flies (N = 3), specimens were sequenced using primers of the cytochrome-b gene (Table 1) [29]. The sequences obtained were analyzed as mentioned above and were then compared with available sequences of sand flies on GenBank.

## Results

### Sand fly fauna composition

**Fig 1:**
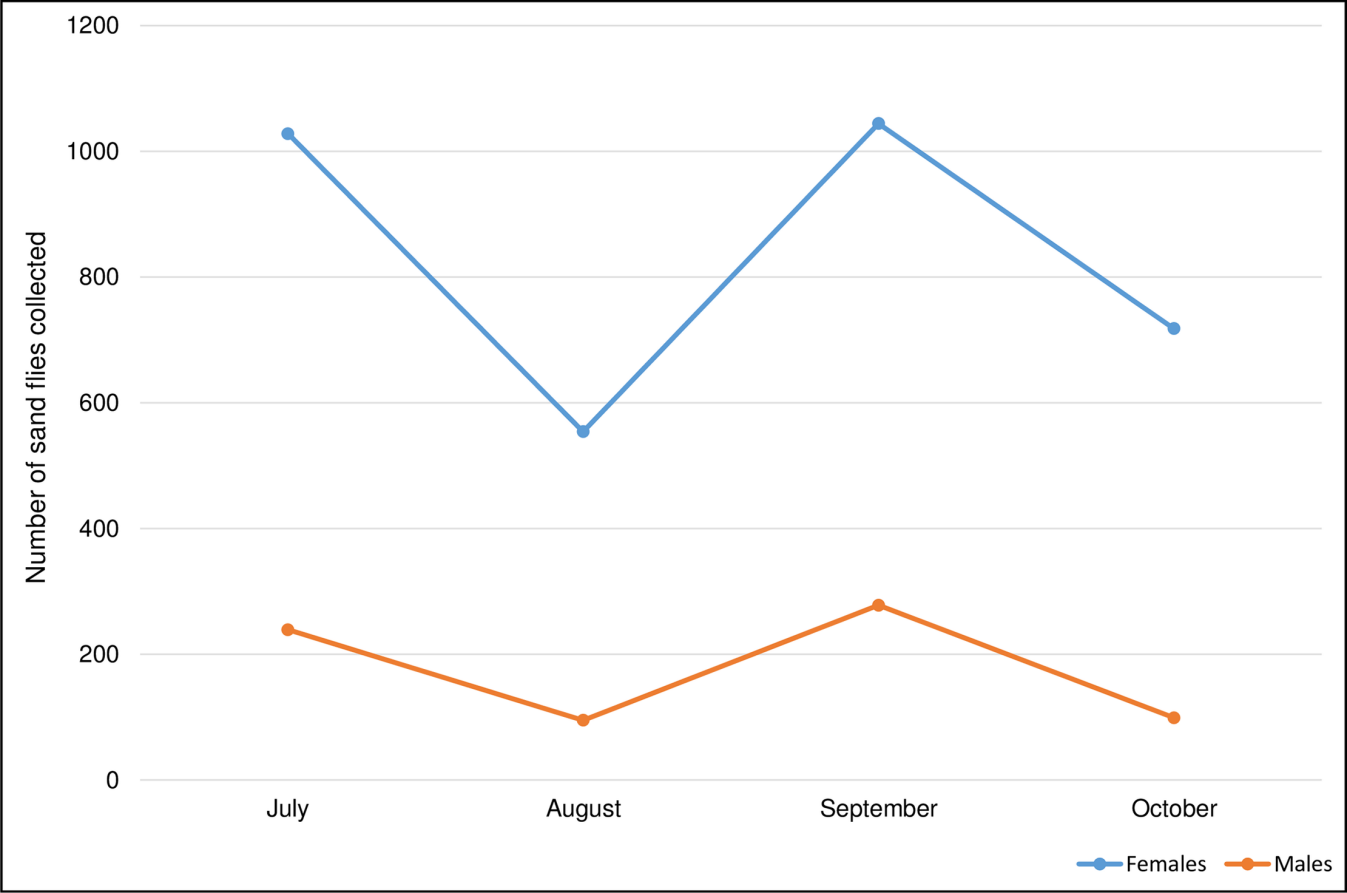
Monthly collections of sand flies by light traps in 2015.

Sand flies were found in all the investigated sites. A total of 4,045 phlebotomine sand flies were collected, of which 3,727 specimens were morphologically identified to species level. The captured sand flies were composed as follow: 2,306 (61.87%) unfed females, 716 (19.21%) fully or partially blood-fed females, and 705 males (18.91%), with a sex ratio of 0.23. The sand fly collection was most abundant in July, with a collection of 1,267 flies (31.32%) and in September with 1,322 (32.68%) (Fig 1).

The phlebotomine species fauna in the studied area is composed of seven species: six belonging to *Phlebotomus* genus of which five belong to *Larroussius* subgenus including *P*. *(L*.*) perniciosus* (1,874; 50.28%), followed by *P*. *(L*.*) perfiliewi* (974; 26.13%), *P*. *(L*.*) longicuspis* (817; 21.92%), *P*. *(L*.*) langeroni* (12; 0.32%) and *P*. *(L*.*) ariasi* (2; 0.05%). One species belonged to *Phlebotomus* subgenus, *P*. *(P*.*) papatasi* (16; 0.42%) and, finally, a single species belonging to *Sergentomya* genus, *Sergentomyia (S*.*) minuta* (32; 0.85%) (Table 2). *P*. *(L*.*) perniciosus* was the most ubiquitous in the four localities monitored and was the predominant species in Bourkika (1495; 79.7%) and Sidi Boufadhel (62; 50%) localities while *P*. *(L*.*) perfiliewi* was the most abundant species in Bouyerssane (764; 54.33%) and El Hadjout (121; 37.46%) (Table 2).

**Table 2:** Sand fly species densities identified by site.

Species	Sandflies by collection site in Tipaza province, *n*								Total		Density, n (%)	
	Bourkika		Bouyerssane		Hadjout		Sidi boufadhel					
	M	F	M	F	M	F	M	F	M	F	
*P*. *(L*.*) perniciosus*	282	1213	54	176	6	81	13	49	355	1519	1874 (50.28%)
*P*. *(L*.*) longicuspis*	65	199	178	212	16	99	11	37	270	547	817 (21.92%)
*P*. *(L*.*) perfiliewi*	4	75	43	721	-	121	1	9	48	926	974 (26.13%)
*P*. *(L*.*) langeroni*	1	-	10	-	-	-	1	-	12	-	12 (0.32%)
*P*. *(L*.*) ariasi*	-	-	2	-	-	-	-	-	2	-	2 (0.05%)
*P*. *(P*.*) papatasi*	4	9	1	1	-	-	-	1	5	11	16 (0.42%)
*S*. *(S*.*) minuta*	11	11	-	8	-	-	2	-	13	19	32 (0.85%)
Total	367	1507	288	1118	22	301	28	96	705	3022	3727

M, male; F, Female.

### *Leishmania* DNA detection in sand flies

Detection of *Leishmania* spp. was carried out using DNA extracts prepared from a total of 740 female sand fly specimens divided into 81 monospecific pools including a total of 600 *P*. *(L*.*) perniciosus* (190 unfed, 410 blood-fed), 91 *P*. *(L*.*) longicuspis* (63 unfed, 28 blood-fed), 39 *P*. *(L*.*) perfiliewi* (22 unfed, 17 blood-fed), 1 unfed *P*. *(P*.*) papatasi* and, finally, 9 *S*. *(S*.*) minuta* (8 unfed, 1 blood-fed).

*Leishmania* spp. DNA was detected by qPCR in two *P*. *(L*.*) perniciosus* pools (1 unfed pool, 1 blood-fed pool) and one *P*. *(L*.*) perfiliewi* pool (blood-fed). The presence of *Leishmania* DNA in the three positive pools was further confirmed by a second specific qPCR that allowed us to identify the *L*. *infantum* parasite. Hence, the three pools assayed displayed positive curves indicating the presence of *L*. *infantum* DNA in these pools.

Individually, screening the DNA of positive sand flies in each pool revealed one positive specimen per pool. Thus, the infection rate was 0.33% (2/600) for *P*. *(L*.*) perniciosus* and 2.56% (1/39) for *P*. *(L*.*) perfiliewi*.

### Blood meal analysis

Blood-meal sources were successfully identified by *COI* gene sequencing from 97.7% (86/88) of the total female sand flies analyzed. Sequencing revealed that specimens belonging to the *Larroussius* subgenus mainly fed on small ruminants (74.11%): *P*. *(L*.*) perniciosus* (36.50%), *P*. *(L*.*) longicuspis (15*.*87%*) and *P*. *(L*.*) perfiliewi (15*.*87%*) were found to have fed on sheep, whereas *P*. *(L*.*) perniciosus* (20.63%), *P*. *(L*.*) longicuspis (7*.*93%*) and *P*. *(L*.*) perfiliewi (3*.*17%*) had fed on goats (Table 3). Interestingly, in this study, human blood was the second feeding source of *Larroussius* subgenus species, with 16.47%. Of the three *Leishmania* positive specimens, two were blood-fed and were found to have fed on human blood (1 *P*. *(L*.*) perniciosus*, 1 *P*. *(L*.*) perfiliewi*). Additionally, the only engorged specimen of *S*. *(S*.*) minuta* was also found to have fed on human blood. Furthermore, eight specimens (9.09%), seven *P*. *(L*.*) longicuspis* and one *P*. *(L*.*) perfiliewi*, were found to have fed on equines (*Equus asinus*). Identification of the blood meal sources failed for two engorged sand fly specimens (2.27%) (Table 3).

**Table 3:** Feeding preferences of blood-fed specimens.

Host	Common Name	*P*. *(L*.*) perniciosus*	*P*. *(L*.*) longicuspis*	*P*. *(L*.*) perfiliewi*	*S (S*.*) minuta*	Total (%)
*Homo sapiens*	Human	4	6	4	1	15 (17,04)
*Ovis aries*	Sheep	23	10	10	-	43 (48,86)
*Capra hircus*	Goat	13	5	2	-	20 (22,72)
*Equus asinus*	Donkey	-	7	1	-	8 (9,09)
Unidentified	-	2	-	-	-	2 (2,27)

### Molecular identification of *Leishmania* infected sand flies

The three *Leishmania* positive specimens identified with molecular tools confirmed the morphological identification. The *cytb* sequences obtained from two *P*. *(L*.*) perniciosus* and one *P*. *(L*.*) perfiliewi* showed 100% identity with *P*. *(L*.*) perniciosus* from Tunisia (JN036742) and *(L*.*) perfiliewi* from Italy (JF766972), respectively.

## Discussion

The main objectives of this study were to identify potential sand fly vectors and *Leishmania* species in the province of Tipaza, northern Algeria, against the backdrop of recent HL cases, with four HVL reported in site of Bourkika in 2014. In the measure of monitoring the risk factors that may promote the spread of the disease, the epidemiological status of *Leishmania* infection in sand flies was investigated from this site. We were also interested in the blood meal preferences of engorged phlebotomine sand flies collected in this region. Entomological techniques to identify sand fly species fauna in the region and molecular tools (PCR and sequencing) were used in the epidemiological study of *Leishmania* infection rates and blood meal identification through field-caught phlebotomine sand flies. Although Algeria is considered as an endemic area for human and canine leishmaniasis, molecular techniques are not often applied in the field epidemiological surveys. These tools could be used as a powerful approach to control leishmaniasis in endemic regions.

From the four monitored sites, seven of the 24 sand flies species already diagnosed in Algeria [7,8] were recorded in this study. These belong to two genera: *Phlebotomus* and *Sergentomya*. Unlike laboratory data, which established that the sex ratio is 1 to emergence [30], our field study found a sex ratio of 0.23 (81.08% females). Biodiversity and sympatric coexistence of these species have revealed to be common in the various different sites. Several factors associated to human activity (agriculture, village urbanization) and climate change (global warming) influence sandflies’ density and abundance [22] as well as bioclimatic characteristics and altitude of the area directly affecting the fauna and the flora [14,31,32].

In our study, the predominant species were *P*. *(L*.*) perniciosus* (50.28%), followed by *P*. *(L*.*) perfiliewi* (26.13%). In the Mediterranean region, including Algeria, these species are considered as competent vectors of *L*. *infantum* [10]. *P*. *(L*.*) perniciosus* is widespread in the Mediterranean basin and is frequently related to humid and sub-humid bioclimatic zones, but is also present in arid and semi-arid zones [2,33]. This species was found to be most abundant in the region of Larbaa Nath Irathen (LNI) in the province of Tizi Ouzou (northern Algeria), the historic foci for human and canine VL due to *L*.*infantum* MON-1 in Algeria [8,14]. Meanwhile, *P*. *(L*.*) perfiliewi* was the second most collected species during a study conducted in August 1989 in a village located in the vicinity of an LNI area [34] (Taourit-Aden, altitude 500 m). This species was incriminated as vector of the dermotropic *L*.*infantum* MON-24 parasite, first in Italy and later in Algeria and Tunisia [34]. *P*. *(L*.*) longicuspis* represents 15.83% of the collected specimens. This species is particularly abundant in arid climatic zones of Sahara and was proposed as a potential vector of autochthonous visceral leishmaniasis (VL) cases in this area. However, *L*. *infantum* DNA was detected from this species in the Kabylia region (LNI) where VL is endemic [10,14,35]. Our findings align with previous entomological studies conducted in northern Algeria sites including Larbaa Nath Irathen [34] and Oum el Bouaghi [36], in which *P*. *(L*.*) perniciosus*, *P*. *(L*.*) perfiliewi* and *P*. *(L*.*) longicuspis* represented 46.92% and 43.45% and 7.37%, and 47.8%, 22.1% and 1.5%, respectively, of the collected phlebotomine sand fly fauna. Within the species belonging to the *Laroussius* subgenus collected in this study, males of *P*. *(L*.*) langeroni* and *P*. *(L*.*) ariasi* were found at very low rates. To our knowledge, only males of *P*. *(L*.*) langeroni* have been successfully collected in Algeria [7,14]. This could be explained by the low number of captured specimens (n ≤ 24) in all field studies conducted until now in Algeria. However, *P*. *(L*.*) ariasi* females have been recorded but considered to be rare [8]. The typical Mediterranean climate of the Tipaza province is appropriate for the spread of the *Larroussius* subgenus. Indeed, previous studies conducted in other northern regions of Algeria have reported the same observation regarding the abundance and distribution of sand flies and most studies correlate their results to the characteristics of the area and climate [8]. *Sergentomyia (S*.*) minuta* and *P*. *(P*.*) papatasi* were found at low levels in this investigation. In Algeria, these two species are mostly related to semi-arid, arid, and Saharan regions, where HZCL is reported [17].

Since vector dispersion is one of the major factors determining the potential rate of pathogen dissemination [1,37], high abundance of *Phlebotomus* genus in the studied area may represent a risk of transmission or even the introduction of non-endemic parasite species from other endemic regions. Indeed, prevalence studies of *Leishmania* infection in phlebotomine vectors are valuable indicators of *Leishmania* transmission intensity [22]. The molecular study of *Leishmania* infection in selected sand flies from Bourkika showed an infection rate of 0.4% (3/740). According to previous reports, a high *Leishmania* infection rates have been observed in fed females compared with unfed females. These latter include probably newly emerged adults, which are expected to be *Leishmania* parasites free before taking blood meals [38,39]. However, in our study, two *Leishmania* positive specimens were unfed while the third was blood-fed.

The individual prevalence by sand fly species showed a low infection rate: 0.33% for *P*. *(L*.*) perniciosus* and 2.56% for *P*. *(L*.*) perfiliewi*, which are the major vectors of *L*. *infantum* in northern Algeria [6]. Generally, in epidemic leishmaniasis communities, the prevalence of *Leishmania* in natural sand fly female populations ranges from 0.7 to 2.0% and seldom exceeds 2% [39,40]. There are many parameters that can affect the prevalence of sand flies with transmissible *Leishmania* parasites, including the number and availability of mammals with infective leishmaniasis [40,22]. In the Kabylia region of northern Algeria, where VL is endemic, previous study showed a low prevalence of 0.2% (1/490) in *P*.*(L*.*) perniciosus* females that were dissected and found to be naturally infected by the viscerotropic *L*. *infantum* zymodeme MON-1 [10]. Meanwhile, one *P*. *perfiliewi* 0.12% (1/801) was found to be naturally infected by the dermotropic *L*. *infantum* MON-24 in the region of Ténès, northern Algeria [10]. However, a recent study conducted in Larbaa Nath Irathen, another VL endemic region in the Kabylia, has reported that the collected *(L*.*) perfiliewi* and *P*. *(L*.*) perniciosus* were all found to be free of *Leishmania* spp. infection [14]. As for other countries of North Africa, the *L*. *infantum* infection rates in *P*. *perfiliewi* and *P*. *perniciosus* sand flies are 0.12% and 0.16% respectively in Tunisia [41]. In north Morocco, *P*. *(L*.*) perniciosus* is the most abundant and proposed as vector of *L*. *infantum* in some endemic areas [42]. In 2018, an epidemiological study conducted in periurban areas situated in North-eastern Morocco revealed the presence of *L*. *infantum* DNA in *P*. *(L*.*) longicuspis* and *P*. *(L*.*) perniciosus* with infection rates of 2.51% and 7.27% respectively [43]. *P*.*(L*.*) longicuspis* has also been found to be infected by *L*. *infantum* in a HL focus in northern Morocco [44]. In the Northern side of the Mediterranean Sea, the global *L*.*infantum* infection rate in sandflies reported over three years of investigation have been evaluated to 3.65% in *P*. *(L*.*) perniciosus* in Spain [22]. In a high-endemic focus of canine leishmaniasis in Rome province, Italy, *Leishmania* infection rate was much higher in sandflies with 47.2% (60/127) while the prevalence of anti-leishmanial antibodies in dogs was 33.3% [45]. A recent study in Serbia, central Europe, showed the emergence of sporadic human and canine leishmaniasis cases in the country which were confirmed by entomological surveillance by the presence of *Leishmania spp*. in sand flies with an infection rate of 4% [46].

In North Algeria as well as in Tipaza province, *L*. *infantum* causes both VL and NCL. According to the data of the public health department of Tipaza, 337 HL (305 NCL, 32 VL) cases were recorded between 2001 and 2014. Furthermore, a recent study reported an outbreak of CL, including infection with *L*. *infantum* and *L*. *killicki* strains from this area [16]. This sympatric existence of multiple *Leishmania* species were also reported from northern-east of Algeria and northern Sahara [15,19]. Our finding highlights the role of *P*. *(P*.*) perniciosus* and *P*. *(P*.*) perfiliewi* in the spread of *L*. *infantum* in the monitored sites because of their high abundance and the detection of the parasite through these two species. Further studies are needed to investigate the presence of *L*. *killicki* vectors and/or any other sympatric existence of *Leishmania* species in the region of Tipaza as well as in other regions of Algeria.

Blood meal analysis to identify haematophagous arthropod feeding sources, other than sand flies and the study of their host preferences, is essential to better understand the transmission dynamics of vector-borne pathogens [47].

In the present study, the molecular identification of blood meal sources was only applied in the region of Bourkika. The results revealed four sources of phlebotomine blood feeding. For the first time in Algeria, this study provided molecular data about the sources of sand flies blood meals in a leishmaniasis focus. The results obtained reveal that small ruminants’ blood was the most common feeding source of *Laroussius* sub-genus sand flies in the region of Bourkika, followed by human blood and then donkey blood. The undetermined blood meal sources were probably due to either the low amount of host blood in the sand flies or the fact that the meal has been partially digested before being collected [33,48]. Specimens collected in this study were mostly caught inside stables, sheep pens where sheepdogs are also present, and close to human habitats. This explains why the majority of sand fly species were found to feed on animals living in these shelters and does not necessarily imply their preference for a particular *Leishmania* susceptible host (domestic dogs) [33,48]. The same observation was reported in a study conducted in a high-endemic focus of canine leishmaniasis in Italy, where a significant number of *P*. *perniciosus* was found to feed on avian and ovine blood related to the habitats where specimens were collected [45]. In contrast, a recent study conducted in Spain showed that *P*. *perniciosus* preferably feeds on rabbits and hares, which seems to play a role of wild reservoirs of leishmaniasis in the area [22]. In Algeria, the role of this species in the transmission of *Leishmania* species has never been investigated but may not be excluded and requires further explorations. Interestingly, the two blood-fed females reported to be positive for *L*. *infantum* in this study, were found to have fed on humans. This important relationship suggests the possible transmission of *L*. *infantum* to humans in this area. The high rate of sand flies blood feeding on animals which are non-reservoirs of *Leishmania* (small ruminants and donkeys) observed in this study may explain their low prevalence for *Leishmania* spp. infection. On the other hand, information obtained on blood-meal sources could explain the high quantity of sand flies found in shelters which represent phlebotomine sand fly breeding sites, due to the plentiful organic soil matter, the natural ecotype usually occupied by immature phlebotomine [49].

### Conclusions

Our epidemiological investigation combines entomological and molecular approaches to update the distribution, *Leishmania* infection status, and blood meal origin of sand flies captured in the region of Tipaza in north-west Algeria. Data reported here revealed the high abundance of *Phlebotomus (Larroussius)* species including major *L*. *infantum* vectors and the detection of the parasite among these latter. Further studies are required for better understand the current state of the disease in Tipaza as well as other affected areas of the country, by introducing innovative and rapid techniques to establish a control program for these diseases.
